# Evaluation of a father and son with atypical chronic myeloid leukemia with *SETBP1* mutations and a review of the literature

**DOI:** 10.1590/1414-431X20154557

**Published:** 2015-05-26

**Authors:** L. Wang, F. Du, H.-M. Zhang, H.-X. Wang

**Affiliations:** 1 Department of Hematology, The Central Hospital of Wuhan, Tongji Medical College, Huazhong University of Science and Technology, Wuhan, China; 2 Department of Gastroenterology, Union Hospital, Tongji Medical College, Huazhong University of Science and Technology, Wuhan, China

**Keywords:** Atypical chronic myeloid leukemia, *SETBP1* mutation, Chronic neutrophilic leukemia, *CSF3R* mutation

## Abstract

We report the case of a father and son diagnosed with atypical chronic myeloid leukemia (aCML). Both patients harbored *SETBP1* mutations, which are present in 24.3% of aCML patients. Moreover, both shared the variant encoding p.Pro737His, but the aCML severity was greater in the son because of the presence of two other missense mutations causing p.Asp868Asn and p.Ser885Arg alterations. *SETBP1* mutations may be associated with an adverse prognosis, so their detection would help in the diagnosis of aCML and the determination of a patient's prognosis.

## Introduction

Atypical chronic myeloid leukemia (aCML), a rare disorder of hematopoietic stem cells, has both myelodysplastic and myeloproliferative characteristics and is classified as an example of myelodysplastic/myeloproliferative neoplasms (MDS/MPN) according to the World Health Organization (WHO) classification system (1). aCML shares clinical and laboratory features with CML, but lacks the *BCR-ABL* fusion gene.

*SETBP1* encodes SET-binding protein 1, a binding partner for the multi-functional SET protein, which is involved in apoptosis, transcription, and nucleosome assembly. A 2013 analysis of exome sequences from 8 aCML cases led to the identification of recurrent *SETBP1* somatic mutations; then targeted resequencing demonstrated the *SETBP1* mutations were present in 24.3% of 70 patients with aCML, as well as in the closely related disorders unclassified MDS/MPN (3/30, 10%) and chronic myelomonocytic leukemia (CMML; 3/82, 4%), and in one of four cases of chronic neutrophilic leukemia (CNL) (2). Among these diseases, CNL was relatively difficult to differentiate from aCML until 2013, because the diagnosis of both diseases required the exclusion of other hematologic neoplasms, as molecularly defined in the WHO Classification of Myeloid Disorders (1). Recently, this situation was resolved because WHO-defined CNL has been shown to be associated with mutations in the gene encoding colony-stimulating factor 3 receptor (*CSF3R*), most commonly *CSF3R* T618I (3–6).

Here, we report two cases of aCML with *SETBP1* mutations in a father and his son.

## Case description

**Figure 1:**
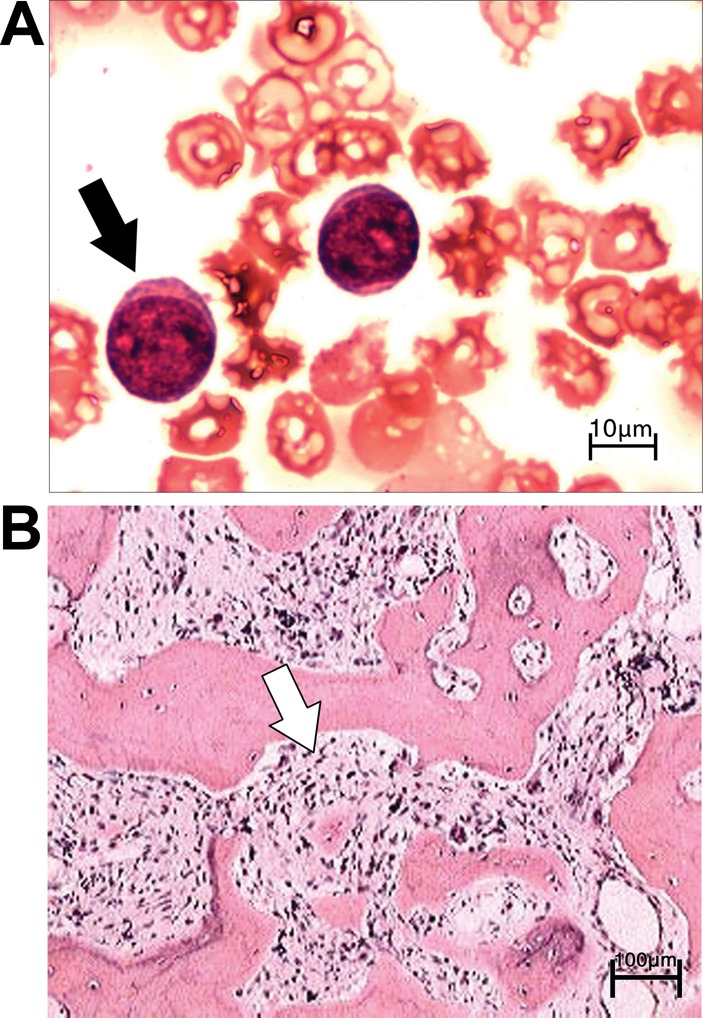
Bone marrow smear and biopsy of the son. *A*, Bone marrow smear stained with Wright-Giemsa (original magnification, 1000×). Black arrow: myeloblast (July 2013). *B*, Bone marrow biopsy stained with hematoxylin and eosin (original magnification, 100×). White arrow: fibroblast proliferation (July 2013).

The 30-year-old son was admitted to our hospital on July 2, 2013, because of an enlarged spleen with a maximum thickness of 9.6 cm. His peripheral blood count status was: white blood cells (WBCs), 16.75×10^9^/L; hemoglobin, 8.4 g/dL; and platelets, 620×10^9^/L. Bone marrow aspiration revealed hypercellularity, different cell body sizes, diminished or moderate cytoplasms, round or oval nuclei, enhanced nuclear chromatin, and 10% myeloblasts. Differential flow cytometric analysis demonstrated that the blasts expressed the myeloid antigens CD34, CD13, and CD117. A bone marrow biopsy showed a small amount of fibroblast proliferation (Figure 1), and conventional chromosomal analysis revealed a normal karyotype (46, XY). Further analysis indicated that *BCR/ABL*, *JAK2* 617F, *JAK2* exon 12, *FIP1L1/PDGFA*, *ETV6-PDGFRB*, *MPL* W515L/K, and c-kit/D816V mutations were absent. However, a *SETBP1* mutation was detected in a SKI homologous region (amino acids 706-917) in the bone marrow sample.

**Figure 2:**
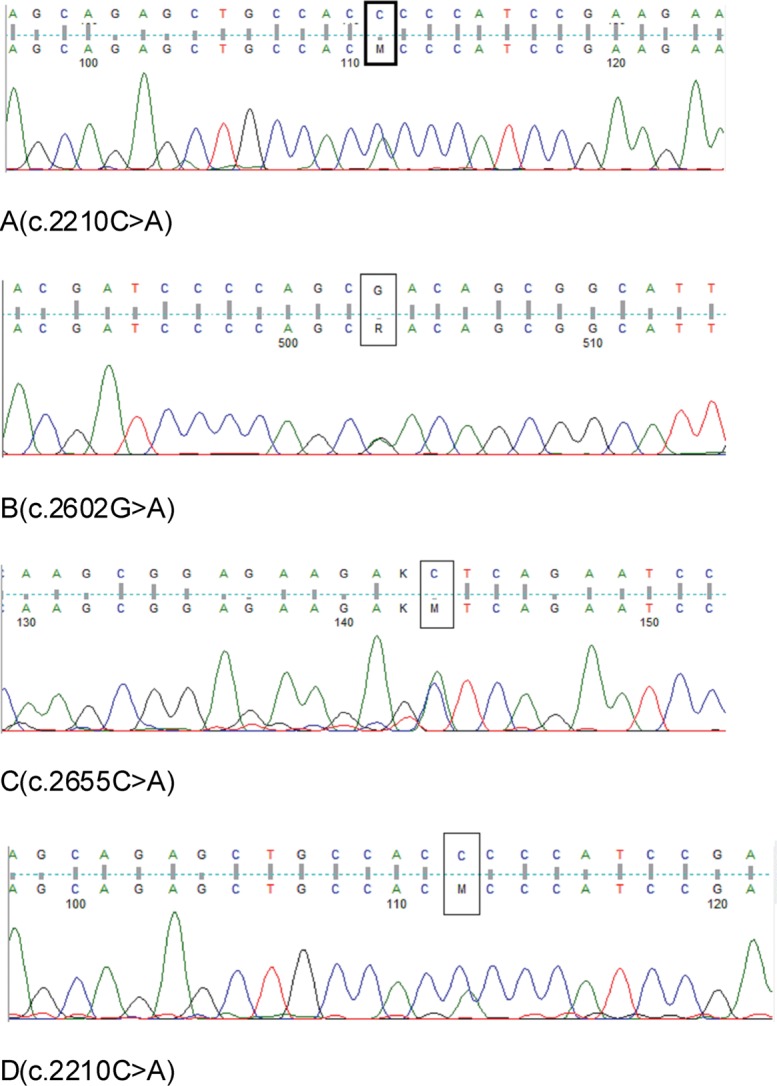
*SETBP1* mutations (boxed) present in the son (*A*-*C*) and the father (*D*). Upper rows indicate normal sequences, and lower rows indicate patient sequences.

PCR and Sanger sequencing to screen for *SETBP1* mutations (using primer pairs primer 1, forward: 5'-GTTGCTCTGAAGGCAAAAGC-3' and reverse: 5'-GTTGTTGTCTGTCCCAATGC-3', and primer 2, forward: 5'-GAAGCTGTCTCCACCCAGAC-3' and reverse: 5'-AGAGCAACGGGTCATACTGG-3') identified three missense mutations causing p.Pro737His, p.Asp868Asn, and p.Ser885Arg alterations (Figure 2).

A low dose of cytarabine (30 mg/day) was administered for 14 days each month beginning July 11, 2013, and interferon α-2a (300 MU) was administered three times weekly by subcutaneous injection but was discontinued because of intolerance after several administrations. However, no significant improvement in fatigue, spleen size, or bone marrow blasts occurred, and the patient suffered aggravated anemia and persistent fever while being treated with antibiotics. On September 13, 2013, the patient began treatment with imatinib mesylate (400 mg/day) (7), which was withdrawn 1 month later and followed by intermittent treatment with cyclophosphamide and hydroxyurea. On November 19, 2013, an ultrasound revealed that the patient’s liver and spleen had further increased in size, and that both of their inferior borders were located in the pelvic cavity. The peripheral blood count status was: WBCs, 136×10^9^/L; hemoglobin, 5.0 g/dL; and platelets, 705×10^9^/L. On December 2, 2013, the patient died from respiratory failure.

The patient’s father was 59 years old in July 2008 when he was diagnosed with aCML. He presented with a giant spleen and the following peripheral blood count status: WBCs, 34.54×10^9^/L; hemoglobin, 115 g/dL; and platelets, 1190×10^9^/L. Ultrasonography and computerized tomography demonstrated a normal liver but an enlarged spleen with a maximum thickness of 8.3 cm, and 7.4 cm under the left rib cage. Tests for BCR/ABL JAK2 617F and CSF3R mutations were negative, although the bone marrow smear and biopsy were indicative of aCML. He was treated with interferon α-2a (3.0 MU/day) plus low-dose cytarabine (20 mg/m^2^ during days 1-10) for approximately 4 months. The regimen was voluntarily discontinued after the symptoms and peripheral blood count status improved. In August 2013, the father was alive and stable, with no indication of disease progression such as fever, anemia, increased blasts, or an enlarged spleen. Analysis of *SETBP1* mutations in DNA from peripheral blood revealed the presence of the variant encoding p.Pro737His, which was shared with his son (Figure 2). No *SETBP1* mutations were detected in the mother. The father died of cerebral infarction in August 2014.

### Compliance with ethics guidelines

All procedures were in accordance with the ethical standards of the responsible committee on human experimentation (institutional and national) and with the Helsinki Declaration of 1975, as revised in 2000 (5). Written informed consent was obtained from all patients included in the study.

## Discussion

In 2010, *SETBP1* mutations were identified as causative in the rare, lethal disorder Schinzel-Giedion syndrome (8). Recently, *SETBP1* mutations have also been identified in aCML and other closely related hematological malignancies, including CNL, CMML, unclassified MDS, MPNs, and secondary AML evolving from MDS at variable frequencies (1.7-25%) (9-13), as shown in Table 1. Piazza et al. (1) reported that aCML patients with *SETBPI* mutations had a worse prognosis (median survival, 22 versus 77 months) and presented with higher WBC counts at the time of diagnosis (median, 81.0 versus 38.5×10^9^/L) compared with aCML cases with wild-type *SETBP1*. Another team showed that *SETBP1*mutations often co-occurred with cytogenetic markers such as -7/del(7q) and i(17)(q10), which were associated with a reduced overall survival time and an increased risk of leukemic evolution from MDS (14).

**Table 1:**
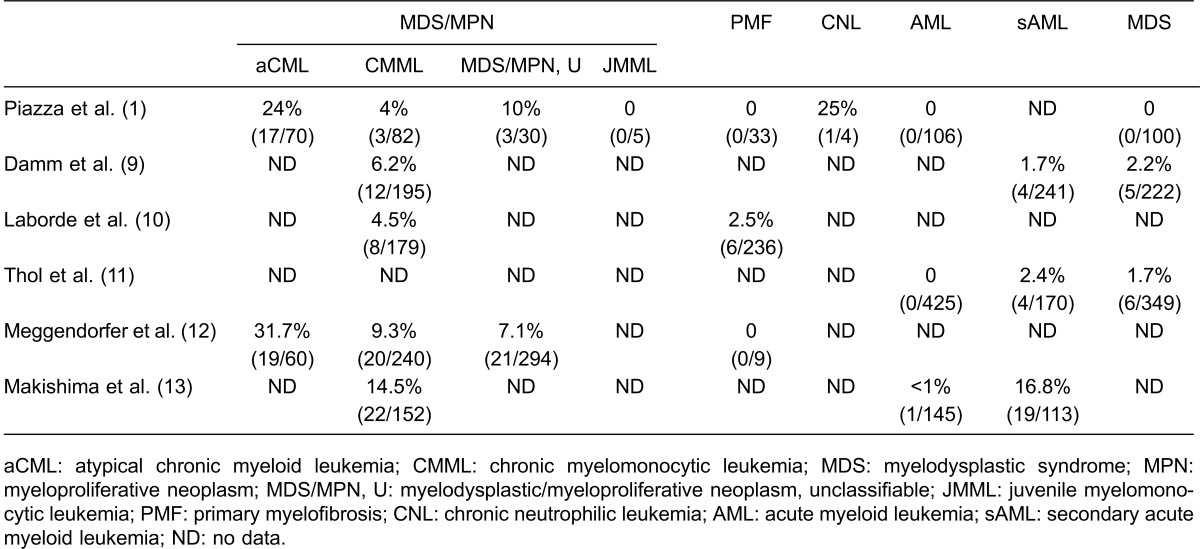
Incidence of SETBP1 mutations in hematological diseases, as reported in the literature.

However, the presence of *SETBP1* mutations was ineffective at differentiating aCML from CNL. *CSF3R* encodes the cell surface transmembrane receptor for granulocyte colony stimulating factor, which induces the proliferation, differentiation, and survival of myeloid progenitors. In 2013, Maxson et al. (3) reported frequent *CSF3R* mutations in patients with CNL (8/9, 88.9%) and aCML (8/18, 44.4%). Two types of *CSF3R* mutation were found: T618I (n=12) and T615A (n=2), which are both located in the membrane proximal region that mediates proliferative and survival signals. Pardanani et al. (4) performed *CSF3R* mutation analysis on 54 patients with clinically suspected CNL (n=35) or aCML (n=19). A central pathology review identified 12 WHO-defined CNL and 9 WHO-defined aCML patients. *CSF3R* T618I mutations were observed in 10 WHO-defined CNL cases, at a mutational frequency of 83% (10/12), and were not seen in WHO-defined aCML, PMF (n=76), or CMML (n=94) cases. A further 3 non-CNL cases also harbored *CSF3R* mutations.

Together, these findings ensure that the diagnosis of CNL is no longer only one of exclusion, and revision of the current WHO diagnostic criteria is expected to include the molecular criterion of *CSF3R* mutation positivity (15). In 2014, Tefferi et al. (5,6) proposed a revision of the WHO criteria, which included major and minor changes in the diagnosis of CNL, such as *i*) a peripheral blood leukocyte level ≥13×10^9^/L, *ii*) a peripheral blood neutrophil/band percentage distribution >80%, and *iii*) the presence of a *CSF3R* T618I mutation or other membrane proximal *CSF3R* mutation. Therefore, although most CNL patients carry *SETBP1* and *ASXL1* mutations, which can also be detected in aCML, *CSF3R* mutations, particularly *CSF3R* T618I, can be used to differentiate them.

In the present cases, we demonstrated that *SETBP1* mutations can co-occur in a father and son with aCML, but it remains to be determined whether these were inherited or acquired. The detection of *SETBP1* and *CSF3R* mutations will play an important role in diagnosing aCML, CNL, and CMML patients, and determining their prognoses. These molecular markers are likely to be incorporated into new diagnostic criteria soon.
